# Increased Urine and Serum Nerve Growth Factor Levels in Interstitial Cystitis Suggest Chronic Inflammation Is Involved in the Pathogenesis of Disease

**DOI:** 10.1371/journal.pone.0044687

**Published:** 2012-09-17

**Authors:** Hsin-Tzu Liu, Hann-Chorng Kuo

**Affiliations:** 1 Department of Urology, Buddhist Tzu Chi General Hospital and Tzu Chi University, Hualin, Taiwan; 2 Institute of Pharmacology and Toxicology, Tzu Chi University, Hualien, Taiwan; University of Sao Paulo Medical School, Brazil

## Abstract

**Objective:**

Interstitial cystitis/bladder pain syndrome (IC/BPS) is considered a bladder disorder due to localized chronic inflammation. This study investigated the nerve growth factor (NGF) levels in serum and urine in patients with IC/BPS.

**Materials and Methods:**

Thirty patients with IC/BPS and 28 normal subjects without lower urinary tract symptoms were recruited from an outpatient clinic. IC/BPS was diagnosed by frequency, bladder pain, and the presence of glomerulations during cystoscopic hydrodistention. Serum and urine were collected before any treatment was given. Serum NGF and urinary NGF/Cr levels were compared between IC/BPS and the controls.

**Results:**

Urinary NGF levels were significantly higher in patients with IC/PBS (26.3±11.2 pg/ml) than in controls (1.40±0.63 pg) (p = 0.014). After normalization, the urinary NGF/Cr levels were significantly greater in IC/BPS (0.69±0.38 pg/mg) than controls (0.20±0.01, p = 0.011). Relative to the levels in control subjects (1.90±0.38 pg/mL), the mean serum NGF levels were higher in patients IC/BPS patients (3.48±0.55 pg/mL) (p = 0.015). No significant correlation was found between the serum and urinary NGF levels in IC/BPS patients. However, the clinical characteristics and medical co-morbidities did not show significant difference between IC/BPS patients with a higher and lower serum NGF level.

**Conclusions:**

Increased urinary NGF levels in IC/BPS patients suggest that chronic inflammation is involved in this bladder disorder. Increased circulating serum NGF levels were noted in over half of patients with IC/BPS, however, the urinary and serum NGF were not inter-correlated and elevated serum NGF did not relate with clinical features.

## Introduction

Interstitial cystitis/bladder pain syndrome (IC/BPS) is a chronic inflammatory disorder of the urinary bladder that manifests as urinary frequency and urgency with or without bladder pain. Several possible etiologies have been proposed for IC/BPS, but no effective long-term treatment has been found. Histological analysis of bladder specimens often shows infiltration of mast cells, suggesting that the disease is mediated by the immune system [1], [2]. Studies of urothelial differentiation in IC/BPS bladder tissue have also demonstrated that acquisition of transitional cell morphology occurred in some of the IC-derived cells, suggesting that in a subset of patients with IC/BPS the differentiation capacity of the urothelium is compromised [3].

Recent investigations into the pathophysiology of IC/BPS have demonstrated elevated levels of several bladder and urinary biomarkers in this bladder disorder, such as nerve growth factor (NGF) [4]–[6]. Results from recent studies suggest a common pathway leading to chronic inflammation in overactive bladder (OAB) and IC/BPS [7], [8]. Tyagi et al. postulated that the increased levels of inflammatory cytokines resulted from complex parasympathetic and peptidergic interactions, which supported the relationship between inflammation and the IC/BPS symptoms of frequency, urgency, and pelvic pain [8].

Urinary NGF is produced from the urothelium and bladder smooth muscles. Patients with idiopathic detrusor overactivity, neurogenic bladder or inflammatory bladder diseases such as IC/BPS have been reported to have increased bladder sensation and urinary NGF levels [4], [9]. NGF is responsible for the growth and maintenance of sensory neurons and appears to play a role in neuroimmune interactions, in tissue inflammation, and in neuroplasticity for neuronal events leading to OAB [10].

Our previous work also found that serum C-reactive protein (CRP) levels increased in IC/BPS as well as OAB patients, suggesting that chronic inflammation plays an important role in the pathophysiology of IC/BPS [7]. Since serum NGF levels are found to increase in several systemic diseases including psychosocial stress, allergy, asthma, and autoimmune diseases [11], [12], measurement of serum NGF in addition to urinary NGF might provide an insight to this mysterious bladder disorder. This study was designed to investigate the levels of serum NGF and urinary NGF levels in patients with IC/BPS.

## Materials and Methods

Patients with IC/BPS and normal subjects without lower urinary tract symptoms (LUTS) were recruited from an outpatient clinic. The diagnosis of IC/BPS was based on the East Asian guideline on IC [13]. Patients should have symptoms of frequency, urgency, bladder pain as well as the presence of glomerulations during cystoscopic hydrodistention performed under general anesthesia. Control subjects were recruited from patients without urological diseases or LUTS and hospital employees.

All patients underwent uroflowmetry, postvoid residual (PVR) urine volume testing, and total bladder capacity measurements. Upon enrolment into the study, serum and urine at full bladder were collected to measure the levels of NGF in serum and urine. The baseline visual analog score (VAS) of pain, maximal bladder capacity and grade of glomerulations after cystoscopic hydrodistention were also recorded. In addition, the medical co-morbidities of patients with IC/BPS were also recorded.

This study was approved by the hospital’s Institutional Review Board. Written informed consent was obtained from all patients and control subjects who participated in this study before serum and urine samples had been collected.

### Urinary NGF Measurement

Urinary and serum NGF levels were measured by the ELISA method [14]. Voided urine was put on ice immediately and transferred to the laboratory for preparation. The samples were centrifuged at 3,000 rpm for 10 minutes at 4°C. The supernatant was separated into aliquots in 1.5-ml tubes and preserved in a freezer at −80°C. At the same time, 3 mL of urine was taken to measure the urinary creatinine level.

NGF concentration was determined using the Emax® ImmunoAssay System (Promega, Madison, WI) with a specific and highly sensitive ELISA kit, which had a minimum sensitivity of 7.8 pg/ml. Assays were performed according to the manufacturer’s instructions. Briefly, NGF level was detected using an antibody sandwich format in 96-well plates. Each well was initially coated with 100 µL of anti-NGF polyclonal antibody and incubated overnight at 4°C, followed by a 1-h incubation with blocking buffer to prevent nonspecific binding. Either 100 µl of urine or 100 µl of NGF standards (0–250 pg/mL) was added to each well followed by incubation for 6 hours at room temperature with shaking. Then the plate was washed, anti-NGF monoclonal antibody was added, and the plate was incubated at 4°C for 14–18 hours. After the plate had been washed, the amount of bound monoclonal antibody was detected using IgG-horseradish peroxidase-conjugated antibody as a tertiary reactant. The unbound conjugate was removed by washing and the plate was then incubated with 100 µL TMB (3,3′5,5′ tetramethyl benzydine) substrate solution for 10 minutes at room temperature. Hydrochloric acid (1 N 100 µL) was added to terminate the reactions. Color change was measured with a Synergy HT Microplate Reader (Bio-Tek Instruments) at 450 nm. The amount of NGF in each urine sample was determined from a standard curve. All samples were run in triplicate and the values were averaged. Total urinary NGF levels were normalized to the concentration of urinary creatinine (NGF/Cr level).

### Serum NGF Milliplex® Assay

Blood (5–10 µL) was withdrawn from each subject and immediately transferred into an anticoagulant vacuum tube. Blood samples were allowed to clot on ice for 30 to 60 min and were then centrifuged in a swinging bucket rotor at 4°C, 3000 rpm for 15 min. The supernatant serum was carefully collected and stored at −80°C until the NGF ELISA testing was performed.

Concentrations of the serum NGF were quantified by using a bead-based human serum adipokine panel B kit (Millipore, Billerica, MA, USA). The Milliplex MAP based on the Luminex xMAP technology by Millipore (Billerica, MA, USA). For the assays, duplicate samples and serial dilutions of the NGF standards were added to 96-well plates and incubated overnight. Data were analyzed by using LX 200™ platforms (Millipore, Billerica, MA, USA). R^2^ of the standard curve is 1.

### Statistical Analysis

Total urinary NGF levels were normalized to the concentration of urinary creatinine (NGF/Cr level). Data on serum NGF and urinary NGF/Cr levels were compared between IC/BPS and the controls. The serum NGF and urinary NGF/Cr data were also compared among IC/BPS patients. Differences in urinary NGF/Cr and serum NGF levels between IC/BPS and controls were compared by the ANOVA (Mann-Whitney test). Furthermore, Pearson’s correlation coefficients were calculated to ascertain correlations between the urine and serum NGF levels. A p-value <0.05 was considered to indicate statistical significance; all tests were two-tailed. All statistical analyses were performed with the statistical package SPSS for Windows (Version 12, SPSS, Chicago, IL).

## Results

A total of 30 patients with IC/BPS and 28 control subjects were enrolled in this study. The mean age was younger in the control (32.4±1.55 years) than IC/BPS (50.6±2.7 years) patients. There were 26 women and 4 men in IC/BPS, whereas 17 women and 11 men in the control group.

**Figure 1:**
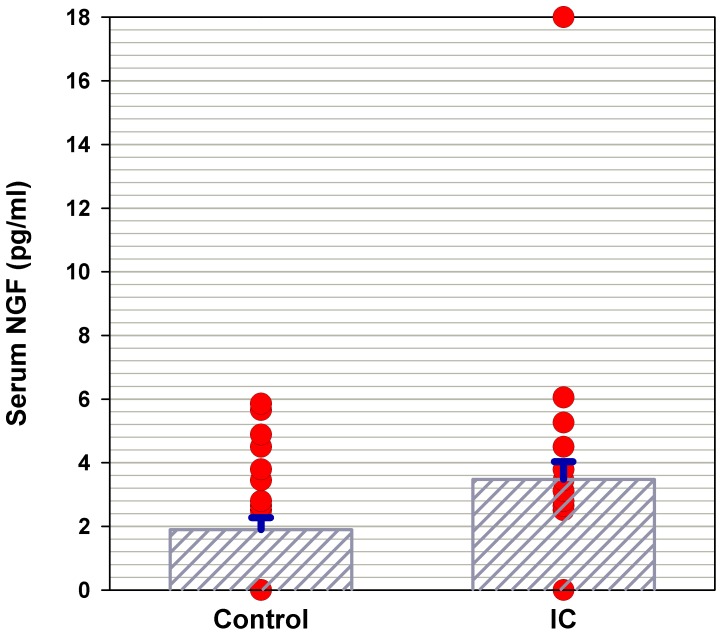
Scattered plot of serum NGF levels in IC/BPS and the control groups. The mean serum NGF levels were significantly higher in IC/BPS patients than in control subjects.

Table 1 lists the urinary NGF, NGF/Cr and serum NGF levels in the control and IC/BPS subgroups. The urinary NGF levels were significantly higher in patients with IC/BPS (26.3±11.2 pg/ml) than in controls (1.40±0.63 pg/ml) (p = 0.014). After normalization, the urinary NGF/Cr levels were significantly greater in IC/BPS (0.69±0.38 pg/mg) than controls (0.02±0.01, p = 0.011). Relative to the levels in control subjects (1.90±0.38 pg/mL), the mean serum NGF levels were higher in patients IC/BPS patients (3.48±0.55 pg/mL) (p = 0.015). Figure 1 shows the scattered plot of serum NGF levels in IC/BPS and the control groups. The mean serum NGF levels were significantly higher in IC/BPS patients than in control subjects.

**Table 1:** Urinary NGF, NGF/Cr, and serum NGF levels between IC/BPS and control patients.

	Control (n = 28)	IC/BPS(n = 30)	P value
Age (range)	32.6±1.56 (22–55 )	51.3±1.87 (22–86)	*p*<0.001
Gender	F:17 M:11	F: 26 M: 4	
Urinary NGF (pg/ml)	1.40±0.63 (0.00–13.6)	26.3±11.2 (0.00–270.4)	P = 0.014
Urinary NGF/Cr (pg/mg)	0.02±0.01 (0.00–0.22)	0.69±0.38 (0.00–9.52)	P = 0.011
Serum NGF (pg/ml)	1.90±0.38 (0.00∼5.85)	3.48±0.55 (0.00–18.0)	P = 0.015

Data are expressed as mean ± standard error.

The medical records of patients with IC/BPS were reviewed to reveal if a higher serum NGF level might appear in patients with autoimmune or allergic disease. The comorbidities of these IC/BPS patients are listed in Table 2 according to serum NGF level. However, we could not find any relationship between serum NGF levels and medical diseases in IC/BPS patients. The patient with very high serum NGF level was found to have multiple medical diseases including peptic ulcer, chronic hepatitis, thyrotoxicosis, reflux esophagitis and lumbar spondylosis.

**Table 2:** The co-morbidities of IC/BPS patients with higher or lower serum nerve growth factor levels.

Co-morbidity	NGF(pg/ml)	Co-morbidity	NGF(pg/ml)
Peptic ulcer, chronic hepatitis, Thyrotoxicosis, reflux esophagitis, spondylosis	18.0	Neurosis	2.79
Migraine	6.05	Affective disorder, breast cancer	2.79
Functional GI disorder	5.26	Function GI disorder, reflux esophagitis	2.79
DM, hypertension	4.5	SLE	2.79
Psychosis	4.5	Cervical cancer, insomnia	2.79
CAD	3.78	Nil	2.79
DM, hypertension	3.78	Nil	2.79
DM	3.44	CAD, neurosis	2.64
Reflux esophagitis	3.44	Nil	2.64
Affective disorder, cervical cancer	3.44	Peptic ulcer, rheumatoid arthritis	2.5
Peptic ulcer, neurosis, migraine	3.44	NPC, fasciitis	2.5
DM, osteoarthritis, dyslipidemia	3.11	Thyroid tumor	2.5
Nil	3.11	CAD, DM, irritable bowel syndrome, dyslipidemia	0
Nil	3.11	Insomnia	0
Nil	3.11	Fasciitis	0

CAD: coronary arterial disease, DM: diabetes mellitus, GI: gastrointestinal, NPC: nasopharyngeal carcinoma, SLE: systemis lupus erythematosus.

There was only 17 IC/PBS patients who had a measurable serum NGF levels (more than 2.5 pg/ml). Analysis of the patients who had serum NGF levels higher than the median, the IC symptom score and pain VAS were not significantly greater than those with serum NGF lower than the median. Although the cystometric bladder capacity was significantly greater in patients with higher serum NGF levels, the maximal bladder capacity, glomerulations degree and voiding frequency all showed no significant difference. (Table 3) The elevated serum NGF and urinary NGF or NGF/Cr were not paralleled in most of the IC/BPS patients. No significant correlation was found between serum NGF and urinary NGF/Cr levels in the IC/BPS or control subgroups.

**Table 3:** Comparison of the clinical characteristics between IC/BPS patients with a higher and lower of median serum NGF levels.

	Lower serum NGF (n = 15)	Higher serum NGF (n = 15)	P value
Age	45.4±9.50	58.7±20.8	0.102
O’Leary-Sant Score	24.1±3.50	23.3±4.03	0.650
VAS pain score	5.0±2.42	4.11±2.32	0.398
Cystometric capacity	200±84.8	292±105	0.035
Qmax	11.3±5.32	13.3±5.92	0.401
PVR	20.4±40.5	62.2±54.3	0.051
Functional capacity	142±81.7	125±96.2	0.665
Frequency	12.7±3.59	11.3±2.87	0.357
Nocturia	4.08±1.55	3.11±1.54	0.165
Maximal bladder capacity	577±128	617±189	0.562
Glomerulation degree	2.08±0.75	1.78±0.83	0.392
Ulcer type case	1	0	
Serum NGF	2.07±1.18	4.13±1.02	<0.0001

Qmax: maximum flow rate, VAS: visual analog score, PVR: postvoid residual, NGF: nerve growth factor.

## Discussion

This study revealed that IC/BPS patients had elevated serum NGF levels compared to the controls. However, although IC/BPS patients also had elevated urinary NGF/Cr levels, the serum NGF levels were not correlated with urinary NGF levels. Patients with higher serum NGF levels did not have more medical diseases than those with lower NGF levels. The IC symptom score and pain VAS were not significantly greater than those with lower serum NGF. These findings suggest that the elevated serum NGF levels in part of IC/BPS patients may be the results of medical co-morbidities rather than cause o IC/BPS, and were not relate with more severe IC symptoms.

NGF is expressed widely in various cells including urothelial cells, smooth muscle cells, and mast cells and can activate the degranulation and proliferation of mast cells. NGF has attracted considerable attention as a key player in the link between inflammation and altered pain signaling. Increased expression of NGF is also present in bladder biopsy specimens from women with IC/BPS [4]. NGF has been implicated as a chemical mediator of pathology-induced changes in C-fiber afferent nerve excitability and reflex bladder activity [14]. The repetitive stimulation of C-fibers by inflammatory stimuli and the upregulation of sensory nerves in the bladder lead to permanent alterations or central sensitization [15]. Endogenous NGF seems to contribute to lower urinary tract dysfunction through regulation of neural function, as well as inflammation and pain [14]. Blockade of NGF using antibody against the NGF receptor prevents neural plasticity and bladder overactivity in experimental animals, suggesting that sequestration of NGF can reduce inflammation and improve OAB or pain symptoms [16].

NGF is also involved in the development and maintenance of specific peripheral and central populations of neuronal cells. NGF may operate through multiple pathways to ultimately regulate physiological homeostasis and behavioral coping [11]. Increased serum NGF levels have been found in vernal keratoconjunctivitis, allergic diseases, and asthma [12]. Increased serum NGF levels might reduce the excitatory threshold of bladder dorsal root ganglia, resulting in increased mechanosensitivity of the bladder wall. Therefore, it is possible that circulating serum NGF levels increase in response to changes in systemic conditions. Elevated levels of circulating NGF might also increase the excitability or susceptibility of sensory receptors in suburothelial nerve fibers such as purinergic receptors P2X3 and transient receptors potential vanilloid receptor subfamily 1 (TRPV1) through intrinsic enhancement, causing the bladder to become more excitable, which in turn leads to symptoms of IC/BPS [17].

Exogenous NGF can induce bladder nociceptive responses and bladder overactivity in rats when applied acutely into the bladder lumen [18] or chronically to the bladder wall or intrathecal space [15]. Increased NGF expression is directly involved in the emergence of bladder-related nociceptive responses in cystitis. It is noteworthy that humans with bladder-associated pain also have a higher incidence of other non-urological disorders such as vulvodynia, fibromyalgia, chronic fatigue syndrome, temporomandibular joint and muscle disorders or irritable bowel syndrome [19]. These observations raise the possibility that several distinct peripheral or central sensory disorders might be activated by nociceptive stimuli in the bladder of patients with IC/BPS. The possible pathway might involve circulating inflammatory proteins such as NGF.

Although serum and urinary NGF levels were demonstrated to increase in IC/BPS patients, not all IC/BPS patients had elevated serum NGF. The elevated serum NGF and urinary NGF levels also show no significant correlation. It is difficult to explain why the serum and urinary NGF levels do not correlate in IC/BPS patients. The most possible cause is that IC/BPS is a syndrome with heterogenous pathogenesis. Elevated urinary NGF level predict that the patients might have chronic inflammation localized to the urinary bladder, whereas patients with elevated serum NGF suggest that some other systemic disorders are also involved in them. In addition, the degree of increased urothelial permeability in IC/BPS bladders might be different. Patients with severe urothelial leakage might have a higher urinary NGF level without a measurable bladder NGF level. On the other hand, patients with a less permeable urothelium might have less urinary NGF level but the bladder NGF levels are elevated which also increase the serum NGF level.

Recently, increasing emphasis has been given to the role of urinary neurotrophins, namely NGF and brain derived neurotrophic factor (BDNF), as key players in some urinary bladder dysfunctions, such as OAB and IC/BPS [20]. NGF has been considered to act as a local stress mediator in perceived stress and allergy and that increased NGF message contributes to worsening of cutaneous inflammation [21]. Stress, glucocorticoids and anti-depressant treatment have been found to modulate the expression of BDNF and NGF [22]. Stress deteriorates bronchial asthma by inducing a pro-inflammatory cytokine profile in allergic asthmatics, including NGF and tumor necrosis factor-alpha [23]. Raised serum NGF levels have been reported as an acute stress reaction and NGF serum concentrations rise after successful cognitive-behavioral therapy of generalized anxiety disorder [24]. Stress has been strongly linked to the pathogenesis of IC/BPS [25]. Irritable bowel syndrome, fibromyalgia and chronic fatigue syndrome are more prevalent in patients with interstitial cystitis/painful bladder syndrome than in asymptomatic control subjects, and result in significant impact [26]. The circulating inflammatory biomarkers such as NGF or BDNF might be the underlying pathophysiology of mixed urological disorders and non-urological diseases. However, whether the elevated serum NGF levels can indicate the presence of chronic stress and inflammation in IC/BPS is not conclusive based on the results of this study. The elevated serum NGF levels may be irrelevant to IC and are the result of medical co-morbidities rather than cause. Therefore, the usefulness of serum NGF as the biomarker for diagnosis or severity of IC/BPS remains undetermined.

NGF-activated mechanisms might be a potential target for treatment of painful symptoms in IC/BPS. Recently, treatment of IC/BPS has focused on anti-NGF antibody administered via subcutaneous injection. Anti-NGF antibody has been shown to decrease the levels of circulating NGF and reduce sensory nerve sensitivity, thereby improving painful bladder symptoms of IC/BPS [27]. If circulating serum NGF is a consequence of systemic disease or localized lesions, the effect of circulating NGF on bladder function can only be eliminated when the diseases have been eradicated. The results of this study, which confirmed that serum NGF levels are increased in part of IC/BPS patients, further enhance the therapeutic possibility of that promising treatment.

The limitation of this study is the lack of age match controls. Because most of the middle aged patients visiting urological department had lower urinary tract symptoms, we could not enroll the age match controls for comparison in this study. A future study with large cohort of IC patients and age matched controls is mandatory.

### Conclusions

The presence of increased serum and urinary NGF levels in patients with IC/BPS suggests that this bladder disorder might be caused by chronic inflammation. Increased serum NGF levels was noted in over half of patients with IC/BPS, however, the urinary and serum NGF levels were not inter-correlated and elevated serum NGF levels did not related with clinical features.
